# Botulinum Toxin Type A Injection Into the Medial Gastrocnemius for Refractory Plantar Fasciitis: A Case Series

**DOI:** 10.7759/cureus.99254

**Published:** 2025-12-15

**Authors:** João Ventura Luís, Gonçalo Martins e Pereira, David Cordeiro, Margarida M Freitas, Sara Lorga

**Affiliations:** 1 Physical and Rehabilitation Medicine, Unidade Local de Saúde de Almada/Seixal - Hospital Garcia de Orta, Almada, PRT

**Keywords:** botulinum toxin type a, chronic plantar fasciitis, medial gastrocnemius, off-label treatment, refractory, ultrasound-guided injection

## Abstract

Plantar fasciitis is the most common cause of pain on the plantar aspect of the hindfoot and may become refractory even after months of conservative care. It is common not only in athletes engaged in activities involving repeated microtrauma to the heel, such as running, walking, basketball or tennis, but also in middle-aged non-athletes with risk factors such as prolonged standing, excessive body weight, foot posture abnormalities and gastrocnemius tightness. Surgical options such as partial plantar fasciotomy or surgical release of the medial gastrocnemius aim to reduce tension in the Achilles-plantar fascia complex, but carry perioperative risks.

We report a single-center three-patient case series evaluating a minimally invasive alternative: ultrasound-guided botulinum toxin type A (BoNT-A) injections into the medial gastrocnemius to reduce calf tone and offload the plantar fascia. Patients included in this study had a clinical diagnosis of chronic plantar fasciitis lasting >12 months and were refractory to multimodal conservative care, including supervised physical therapy, extracorporeal shockwave therapy, orthoses or prior corticosteroid injections. These patients were selected for the injection of two motor-point targets within the medial gastrocnemius with 50 U of BoNT-A under ultrasound guidance. A standardized post-injection program emphasized calf and plantar-fascia stretching, progressive eccentric calf strengthening, intrinsic foot activation and gradual return to activity. Outcomes were the Numeric Rating Scale (NRS) for pain, the Foot and Ankle Disability Index - Activities of Daily Living Subscale (FADI-ADL) and the Maryland Foot Score (MFS) at baseline and follow-up at four weeks post-injection. The index patient (male, 66 years) improved from NRS 7/10, FADI-ADL 76, MFS 78 to NRS 3/10, FADI-ADL 99, MFS 88 at four weeks. Case 2 (female, 57 years) improved from NRS 9/10, FADI-ADL 39, MFS 33 to NRS 4/10, FADI-ADL 60, MFS 74 at four weeks. Case 3 (female, 45 years; bilateral symptoms) improved from NRS 9/10, FADI-ADL 77, MFS 76 to NRS 7/10, FADI-ADL 81, MFS 80 at four weeks. No complications occurred, including no detectable plantar flexion weakness.

These cases suggest that targeted BoNT-A injection into the medial gastrocnemius may reduce pain and improve function in refractory plantar fasciitis while avoiding surgery. Controlled studies are warranted to define optimal dosing, injection sites and rehabilitation protocols, and also to compare gastrocnemius-targeted injection with corticosteroid and local anesthetic injection, extracorporeal shockwave therapy and surgery.

## Introduction

Definition and epidemiology

Plantar fasciitis is a degenerative, overload-related disorder of the plantar fascia origin at the medial calcaneal tuberosity rather than an acute inflammatory process [1]. It is the most common cause of plantar heel pain in adults and affects both athletes exposed to repetitive heel micro-trauma and non-athletes with occupational or lifestyle risk factors [2,3]. Typical risk factors include elevated body mass index, prolonged standing or walking, foot posture abnormalities (e.g., pes planus) and limited ankle dorsiflexion related to gastrocnemius tightness. Improper footwear, such as shoes with inadequate arch support, minimal cushioning, excessive heel height or poor stability, can increase mechanical stress on the plantar fascia and contribute to the development of plantar fasciitis [4,5].

Biomechanical rationale

The plantar fascia contributes to longitudinal arch support through the windlass mechanism: when the hallux dorsiflexes, the fascia tightens, the arch elevates and the foot stiffens for propulsion [6,7]. Because the fascia is mechanically coupled to the Achilles-gastrocnemius-soleus complex across the calcaneus, isolated gastrocnemius tightness increases plantar fascial strain in mid- to terminal-stance [8,9]. Reducing calf tendon tension can therefore offload the plantar fascia.

Clinical presentation

Patients typically report sharp, localized pain at the plantar-medial heel, worse with the first steps after rest and after prolonged standing or walking. Examination demonstrates point tenderness over the medial calcaneal tuberosity and pain with passive ankle dorsiflexion or toe extension. Ankle dorsiflexion is often more restricted with the knee extended than flexed, suggesting gastrocnemius predominance, also known as a positive Silfverskiold test. Neurologic examination is normal, and other causes of heel pain should be considered when atypical features are present [1].

Conservative management

First-line care is multimodal and includes activity modification and load management, plantar-fascia-specific and calf stretching, custom-molded foot orthoses, night splints (ankle dorsiflexion and/or hallux extension), manual therapy, progressive strengthening of the calf and intrinsic foot muscles, education on footwear and gradual return to activity [1,4]. Common adjunctive treatments include extracorporeal shockwave therapy and image-guided injections, most frequently with corticosteroid and local anesthetic [10,11]. Responses vary, and a substantial subset remain symptomatic after six to nine months of structured care [12].

Surgical options and indications

When pain persists despite exhaustive conservative treatment, surgical options are considered: partial plantar fasciotomy (open or endoscopic) to release a limited portion of the fascia and proximal medial gastrocnemius release to address equinus and reduce Achilles-plantar fascia tension. Surgery is generally reserved for patients with disabling symptoms beyond 6-12 months, consistent examination findings and appropriate imaging or exclusion of alternative diagnoses. Risks include arch instability with over-release, nerve irritation and prolonged recovery [13,14].

Role of botulinum toxin A (BoNT-A)

BoNT-A induces a reversible chemo-denervation and tone reduction in the target muscle. Injecting the medial gastrocnemius under ultrasound guidance aims to decrease calf-tendon tension and, by mechanical coupling, offload the plantar fascia, putting into practice the same rationale as gastrocnemius release while avoiding operative morbidity. In this context, BoNT-A may be considered for carefully selected patients with chronic symptoms, documented with gastrocnemius tightness and failure of comprehensive conservative therapy [5,15-17]. Despite a plausible biomechanical rationale, clinical evidence supporting ultrasound-guided injection of BoNT-A into the medial gastrocnemius as a non-surgical treatment for chronic plantar fasciitis remains very limited and based on a small number of randomized studies with modest sample sizes. Therefore, available data should be considered preliminary, and our findings contribute to additional evidence for this potential therapeutic option [16,17].

Outcome measures

We assessed treatment response using three validated and widely used outcome measures, each capturing a different clinically relevant domain.

Numeric Rating Scale (NRS)

To evaluate pain, we used the NRS, an 11‑point patient-reported numeric scale anchored at 0 as “no pain” and 10 as “worst imaginable pain.” Patients rated their average pain over the preceding week [18].

Foot and Ankle Disability Index - Activities of Daily Living Subscale (FADI-ADL)

In order to assess for symptoms and functional limitations relevant to the foot and ankle, we applied the FADI-ADL. The FADI is a patient‑reported questionnaire, covering the past week, and is composed of an ADL with 26 items and a sports subscale with eight items. In this study, we only administered the ADL subscale. The FADI-ADL uses Likert‑type responses scored 0-4 per item (0 = unable/extreme difficulty; 4 = no difficulty or no pain), with an N/A option when a limitation is unrelated to the foot or ankle. The subscale total ranges from 0 to 104 (higher equals better function/less disability). Items encompass standing, walking on even/uneven ground, hills and stairs, curbs, squatting, sleep disturbance, toe‑rise, initial steps, timed walking tolerances, home/ADL/personal care, work demands, recreational activities and pain at rest/during activity/first thing in the morning. In this series, we report raw totals [19].

Maryland Foot Score (MFS)

For evaluation of overall foot status after injury, we implemented the MFS, which is a clinician- and patient-informed composite index. It includes two main domains: pain (scored from “no pain, including during sports” to “disabled,” contributing up to 45 points) and function (up to 55 points), which encompasses walking distance, perceived stability/giving way, need for external support, limp severity, shoe tolerance, and other functional demands of the foot. These components are summed to a total score from 0 to 100 points, with higher scores indicating less pain and better overall foot function. In this study, we report raw total scores, with higher scores reflecting better overall foot status [20].

## Case presentation

Case 1

Case 1 was a 66 year-old male, retired from the navy, with a past medical history of overweight (83 kg at 1,65 m) and arterial hypertension, whose main form of physical activity consisted of daily walks, presenting with chronic right plantar heel pain consistent with plantar fasciitis and refractory to prolonged conservative management. Symptoms had been present for 14 months and were exacerbated by weight-bearing activities, particularly the first steps in the morning and prolonged standing or walking. On physical examination, there was localized tenderness at the medial calcaneal tuberosity. Ankle dorsiflexion, especially with the knee extended, reproduced his heel pain, which was consistent with isolated gastrocnemius tightness. Passive toe extension also elicited plantar fascia-type pain. Podoscopic assessment showed a tendency toward pes planus. Ultrasound confirmed the diagnosis of plantar fasciitis, with plantar fascia thickness exceeding 4 mm. Baseline outcome measures were NRS 7/10, FADI-ADL 76/104 and MFS 78/100.

He had undergone extensive conservative management, including multiple supervised physiotherapy programs focusing on calf and plantar fascia-specific stretching, manual therapy, progressive strengthening and physical modalities. Specifically, he completed four courses of 12 supervised physiotherapy sessions (therapeutic exercise, iontophoresis with a non-steroidal anti-inflammatory drug and ultrasound therapy) and a course of five sessions of radial extracorporeal shockwave therapy, performed weekly. He was already using custom-molded foot orthoses and had previously received one ultrasound-guided corticosteroid and local anesthetic injection, which did not result in sustained benefit. Despite these measures, he remained significantly symptomatic at presentation for BoNT-A injection.

He then underwent an ultrasound-guided chemodenervation protocol targeting the medial gastrocnemius of the right limb. Under aseptic conditions, 50 units of BoNT-A were injected into the medial gastrocnemius, divided across two motor-point targets, using a 27-gauge needle, with negative aspiration performed prior to each deposit. Plantar flexion strength (manual testing and single-leg heel-rise), gait and local reactions at the injection site (pain, bruising/hematoma, infection) were assessed immediately and four weeks after the procedure. He was also questioned about systemic symptoms (e.g., generalized weakness, flu-like symptoms). No immediate or delayed procedural complications were identified, and no clinically detectable plantar flexion weakness was observed.

A structured rehabilitation plan began immediately after the injection. The program emphasized gentle gastrocnemius-soleus stretching with the knee extended (to address isolated gastrocnemius tightness), plantar fascia-specific stretching, progressive eccentric-concentric strengthening of the calf and intrinsic foot muscles and continued use of his custom orthoses.

Outcome measures were collected at baseline and at four weeks post-injection. At four weeks, pain intensity decreased from NRS 7/10 to 3/10. Functional scores improved from FADI-ADL 76/104 to 99/104 and from MFS 78/100 to 88/100. Clinically, he reported a meaningful reduction in activity-related pain and improved walking tolerance, without new weakness or other adverse effects.

Case 2

Case 2 was a 57-year-old female working as an operational assistant in a hospital, on medical leave at the time of assessment, with a medical history of arterial hypertension, severe asthma, hypothyroidism, depressive disorder, fibromyalgia and overweight (72 kg at 1,58 m). She presented with chronic right plantar heel pain, consistent with plantar fasciitis and refractory to prolonged conservative management. Symptoms had been present for 12 months and were aggravated by the first steps in the morning and prolonged standing or walking.

On examination, physical findings were overlapping with those observed in Case 1. She presented with localized tenderness at the medial calcaneal tuberosity, a positive Silfverskiold test and passive toe extension also elicited plantar fascia-type pain. Podoscopic assessment also showed a tendency toward pes planus. Plantar fasciitis diagnosis was again confirmed by ultrasound. Baseline scores were NRS 9/10, FADI-ADL 39/104 and MFS 33/100.

Her prior management included extensive conservative treatment similar to Case 1. She completed four courses of 12 supervised physiotherapy sessions and a course of five sessions of radial extracorporeal shockwave therapy. She had been using custom-molded foot orthoses for the previous four months but had not received a prior corticosteroid injection and had not used a night splint. Despite this prolonged conservative management, she remained significantly symptomatic at the time of referral for BoNT-A injection.

She underwent the same ultrasound-guided chemodenervation protocol as Case 1. No immediate or delayed complications, including plantar flexion weakness, were detected. Post-injection, she followed the same structured rehabilitation program as described for Case 1, starting immediately after the procedure.

At four weeks post-injection, outcomes showed clinically relevant improvement. Pain decreased from NRS 9/10 at baseline to 4/10, FADI-ADL improved from 39/104 to 60/104 and MFS improved from 33/100 to 74/100. She reported better tolerance of prolonged standing and walking, with no post-injection calf weakness or other adverse events.

Case 3

Case 3 was a 45-year-old female who worked in a warehouse but was on medical leave due to her condition and the resulting functional limitations. She had no other relevant past medical history. She presented with chronic bilateral plantar heel pain, clinically similar on both sides and consistent with plantar fasciitis. Symptoms had been present for approximately 15 months and were exacerbated by the first steps in the morning and by prolonged standing or walking.

On physical examination, ankle dorsiflexion, particularly with the knee extended, elicited heel pain bilaterally, compatible with isolated gastrocnemius tightness, and plantar fascia-type pain was reproduced with passive toe extension. Unlike Cases 1 and 2, podoscopic assessment showed a neutral foot arch. Localized point tenderness was present at the medial calcaneal tuberosity on both sides. Ultrasound examination confirmed bilateral plantar fasciitis. Baseline scores were NRS 9/10, FADI-ADL 77/104 and MFS 76/100.

As with the previous cases, she had undergone extensive conservative treatment before BoNT-A injection. This also included four courses of 12 supervised physiotherapy sessions, a course of five sessions of radial extracorporeal shockwave therapy, custom-molded orthoses and a night splint. In addition, she had previously received a corticosteroid and local anesthetic injection without ultrasound guidance at another center, which did not provide sustained benefit. She remained significantly symptomatic at presentation for BoNT-A treatment.

She underwent the same ultrasound-guided chemodenervation protocol, applied bilaterally, with 50 units of BoNT-A being injected into the medial gastrocnemius of each limb (50 units per side). No immediate or delayed adverse events or clinically detectable plantar flexion weakness were observed following bilateral injection. The post-injection rehabilitation plan was identical to that used in the previous cases and began immediately after the procedure.

At four weeks post-injection, there was a modest but clinically relevant improvement. NRS decreased from 9/10 to 7/10, FADI-ADL improved from 77/104 to 81/104 and MFS from 76/100 to 80/100. She reported a reduction in the severity of first-step pain in the morning and easier prolonged standing. No adverse events, including no subjective or objective plantar-flexion weakness, were observed after bilateral gastrocnemius injection.

Table 1 summarizes the key clinical features of the three cases of chronic plantar fasciitis. For each patient, we present demographic data, symptom duration, baseline pain and function scores, prior conservative management, details of the ultrasound-guided medial gastrocnemius BoNT-A injection, and short-term outcomes (NRS, FADI-ADL, MFS).

**Table 1:** Overview of patient characteristics, intervention and outcomes Categorical variables are expressed as counts. B: baseline; F: follow-up; ESWT: extracorporeal shockwave therapy; IU: International Units; BoNT-A: botulinum toxin type A; OnaBoNT-A: onabotulinumtoxin type A; PT: physical therapy; US: ultrasound; NRS: Numeric Rating Scale; FADI-ADL: Foot and Ankle Disability Index - Activities of Daily Living Subscale; MFS: Maryland Foot Score

Case	Age/sex	Duration (months)	Prior treatments	BoNT-A brand, total dose and injection points	NRS		FADI-ADL		MFS	
					B	F	B	F	B	F
1	66/Male	14	PT (4x12); ESWT x5; corticosteroid injection; orthoses	OnaBoNT-A 50 IU injected at two points in the medial gastrocnemius	7	3	76	99	78	88
2	57/Female	12	PT (4x12); ESWT ×5; orthoses	OnaBoNT-A 50 IU injected at two points in the medial gastrocnemius	9	4	39	60	33	74
3	45/Female	15	PT (4×12); ESWT ×5; orthoses; night splint; corticosteroid injection (no US)	OnaBoNT-A 50 IU injected at two points in the medial gastrocnemius	9	7	77	81	76	80

## Discussion

This case series illustrates a minimally invasive approach to refractory plantar fasciitis that targets the gastrocnemius to reduce plantar fascial strain via temporary tone reduction. The biomechanical link between the Achilles tendon-gastrocnemius complex and the plantar fascia provides the rationale for both surgical release of the medial gastrocnemius and non‑surgical chemo‑denervation [16]. In these three cases, a single injection of 50 UI BoNT-A into the medial gastrocnemius yielded clinically meaningful pain and functional gains without weakness or complications.

Beyond the quantitative improvements observed in the outcome scales, these changes translated into clinically meaningful functional gains. In Case 1, pain reduction resulted in pain-free gait with only minimal post-activity discomfort (NRS 2/10) after walking, which was particularly relevant given that his main form of physical activity consisted of daily walks. Additionally, there was resolution of the previously reported morning pain and stiffness. In Case 2, the improvement in symptoms was sufficient to allow discontinuation of medical leave and return to work, with daytime pain reported as NRS 2/10. By contrast, Case 3 showed a more modest clinical response, with persistence of symptoms similar to those at baseline, although with a clearly reduced intensity.

Evidence from randomized and comparative studies suggests that BoNT‑A can improve pain and function in chronic plantar fasciitis, whether injected into the plantar fascia or into the calf muscles. Emerging meta‑analyses report statistically and clinically significant improvements versus control conditions at short‑ to mid‑term follow‑up, although heterogeneity in targets, doses and outcome measures limits firm conclusions [15,16]. In parallel, surgical options, such as partial plantar fasciotomy and gastrocnemius release, may provide relief for selected patients, but involve operative risk, potential arch instability if release exceeds recommended proportions and variable long‑term durability [13,14]. A reversible, ultrasound‑guided BoNT‑A injection provides an intermediate option that aligns with the mechanical off‑loading principle while avoiding surgery.

Patient selection and technique

We recommend confirming isolated gastrocnemius tightness using dorsiflexion testing with the knee extended versus flexed, screening for contributing biomechanical factors (e.g., pes planus) and ensuring a comprehensive trial of high‑load plantar fascia‑specific strengthening with calf stretching and appropriate orthoses. Before proceeding with this treatment modality, contraindications to botulinum toxin, such as myasthenia gravis or pregnancy, must be excluded. In addition, neurological disorders causing gait abnormalities should be ruled out. Finally, it is essential to confirm the diagnosis of plantar fasciitis and exclude other potential causes of pain in the same region, such as Baxter’s nerve neuropathy, plantar fibromatosis (Ledderhose disease), tarsal tunnel syndrome, heel fat pad atrophy, partial plantar fascia rupture or calcaneal fracture.

Ultrasound guidance facilitates accurate targeting and mitigation of neurovascular injury. In our experience, 50 UI of BoNT-A divided across two motor‑point targets in the medial gastrocnemius was well tolerated. However, optimal dosing may vary with muscle size and chronicity.

Limitations

This is an uncontrolled case series with short‑term follow‑up and potential placebo effects. Future randomized trials should compare calf‑targeted BoNT‑A with plantar fascia injections with corticosteroids/local anesthetics or platelet‑rich plasma, extracorporeal shockwave therapy, surgery and define standardized rehabilitation protocols.

## Conclusions

Ultrasound-guided BoNT-A injection into the medial gastrocnemius appears to be a feasible and safe minimally invasive option for chronic, refractory plantar fasciitis, yielding clinically meaningful short-term improvements in pain and function without plantar flexion weakness. By temporarily reducing gastrocnemius tone, this approach applies the same off-loading rationale as medial gastrocnemius release while avoiding operative morbidity and allowing immediate continuation of rehabilitation. In care pathways, gastrocnemius-targeted BoNT-A may serve as a bridge or alternative for patients who have exhausted structured conservative therapy and are reluctant to proceed to surgery.

Future studies should define optimal patient selection (e.g., isolated gastrocnemius tightness, unilateral vs. bilateral disease), dose/dilution and number of injection points and standardized post-injection rehabilitation. Randomized controlled trials are warranted to compare this strategy with intrafascial injection, corticosteroid injection, extracorporeal shockwave therapy, platelet-rich plasma and surgical treatments. These trials should include at least 6-12 months of follow-up and use core outcome measures such as NRS, FADI-ADL and MFS. Health-economic analyses and formal safety surveillance will help clarify the role of this off-label intervention in routine practice.
